# Deep morphological analysis of muscle biopsies from type III glycogenesis (GSDIII), debranching enzyme deficiency, revealed stereotyped vacuolar myopathy and autophagy impairment

**DOI:** 10.1186/s40478-019-0815-2

**Published:** 2019-10-28

**Authors:** Pascal Laforêt, Michio Inoue, Evelyne Goillot, Claire Lefeuvre, Umut Cagin, Nathalie Streichenberger, Sarah Leonard-Louis, Guy Brochier, Angeline Madelaine, Clemence Labasse, Carola Hedberg-Oldfors, Thomas Krag, Louisa Jauze, Julien Fabregue, Philippe Labrune, Jose Milisenda, Aleksandra Nadaj-Pakleza, Sabrina Sacconi, Federico Mingozzi, Giuseppe Ronzitti, François Petit, Benedikt Schoser, Anders Oldfors, John Vissing, Norma B. Romero, Ichizo Nishino, Edoardo Malfatti

**Affiliations:** 1 APHP, Department of Neurology, Raymond Poincaré Hospital, Garches, France; 2 Centre de Référence de Pathologie Neuromusculaire Nord-Est-Ile-de-France, Garches, France; 3 U 1179 INSERM, Université Versailles Saint Quentin en Yvelines; Paris-Saclay, Saint-Quentin-en-Yvelines, France; 4 Department of Neuromuscular Research, National Institute of Neuroscience, National Center of Neurology and Psychiatry, Tokyo, 187-8502 Japan; 5 Centre de Pathologie et Neuropathologie Est, Hospices Civils de Lyon-Lyon 1; Université Claude Bernard Lyon, Institut NeuroMyogène, CNRS UMR 5310 - INSERM U1217, Lyon, France; 6 INTEGRARE, Genethon, Inserm, Univ Evry, Université Paris-Saclay, 91002 Evry, France; 7 Centre de référence de Pathologie Neuromusculaire Paris-Est, Institut de Myologie, GHU La Pitié-Salpêtrière, Assistance Publique-Hôpitaux de Paris, Paris, France; 8 Unité de Morphologie Neuromusculaire, Institut de Myologie, Groupe Hospitalier Universitaire La Pitié-Salpêtrière, Paris, France; 9 Department of Pathology, Institute of Biomedicine, University of Gothenburg, Gothenburg, Sweden; 10 Copenhagen Neuromuscular Center, Department of Neurology, Rigshospitalet, University of Copenhagen, Copenhagen, Denmark; 11 APHP, Hôpitaux Universitaires Paris Sud, Hôpital Antoine Béclère, Centre de Référence des Maladies héréditaires du Métabolisme Hépatique, and Paris Sud University, Clamart, France; 12 Internal Medicine Department Neuromuscular and Inherited Metabolic Disorders Research Laboratory Hospital Clínic de Barcelona, Barcelona, Spain; 13 Centre de référence des maladies neuromusculaires Nord/Est/IdF, Service de Neurologie, CHU Strasbourg, Strasbourg, France; 14 Peripheral Nervous System & Muscle Department, CHU Nice, Université Côte D’Azur, Institute for Research on Cancer and Aging of Nice (IRCAN), INSERM U1081, CNRS UMR 7284, Faculty of Medicine, Université Côte d’Azur (UCA), Nice, France; 15 Department of Genetics and Cytogenetics, AP-HP, Antoine Béclère University Hospital, University Paris Sud, Paris, France; 16 Friedrich-Baur-Institut Neurologische Klinik, München University, Munich, Germany; 17 Université Pierre et Marie Curie- Paris 6, Centre de Recherche en Myologie, UM 76, CNRS, UMR 7215, Institut de Myologie, Paris, F-75013 France

**Keywords:** Glycogen storage disease III, Muscle glycogenosis, Metabolic myopathies, Myopathology, Autophagy, Autophagic impairment

## Abstract

Glycogen storage disorder type III (GSDIII), or debranching enzyme (GDE) deficiency, is a rare metabolic disorder characterized by variable liver, cardiac, and skeletal muscle involvement. GSDIII manifests with liver symptoms in infancy and muscle involvement during early adulthood. Muscle biopsy is mainly performed in patients diagnosed in adulthood, as routine diagnosis relies on blood or liver GDE analysis, followed by *AGL* gene sequencing. The GSDIII mouse model recapitulate the clinical phenotype in humans, and a nearly full rescue of muscle function was observed in mice treated with the dual AAV vector expressing the GDE transgene.

In order to characterize GSDIII muscle morphological spectrum and identify novel disease markers and pathways, we performed a large international multicentric morphological study on 30 muscle biopsies from GSDIII patients. Autophagy flux studies were performed in human muscle biopsies and muscles from GSDIII mice. The human muscle biopsies revealed a typical and constant vacuolar myopathy, characterized by multiple and variably sized vacuoles filled with PAS-positive material. Using electron microscopy, we confirmed the presence of large non-membrane bound sarcoplasmic deposits of normally structured glycogen as well as smaller rounded sac structures lined by a continuous double membrane containing only glycogen, corresponding to autophagosomes. A consistent SQSTM1/p62 decrease and beclin-1 increase in human muscle biopsies suggested an enhanced autophagy. Consistent with this, an increase in the lipidated form of LC3, LC3II was found in patients compared to controls. A decrease in SQSTM1/p62 was also found in the GSDIII mouse model.

In conclusion, we characterized the morphological phenotype in GSDIII muscle and demonstrated dysfunctional autophagy in GSDIII human samples.

These findings suggest that autophagic modulation combined with gene therapy might be considered as a novel treatment for GSDIII.

## Introduction

Muscle glycogenoses are rare inherited conditions presenting with muscle symptoms sometimes associated with other organ involvement. Glycogen storage disease type III (GSDIII) is caused by autosomal recessive mutations in the *AGL* gene encoding the glycogen debranching enzyme (GDE or amylo-alpha-1,6-glucosidase, EC no. 3.2.1.33, UniProt P35573). GDE is an enzyme with two catalytic sites involved in the conversion of cytosolic glycogen to glucose [19].

Clinically, GSDIII is a biphasic disorder. During childhood, patients present a liver metabolic disorder with hepatomegaly and severe fasting hypoglycemia, hyperlipidemia, and hyperketonemia. During adolescence and adulthood, patients develop a progressive myopathy that can be accompanied by muscle weakness and exercise intolerance [3, 20]. In this phase, the metabolic impairment is less prominent and the patients are referred to muscle specialists [3–9]. A minor percentage (15%) of patients develop cardiomyopathy [21] and other liver complications such as cirrhosis. Hepatocellular adenomas (HCA) and carcinomas (HCC) have previously been described in GSDIII patient [9].

From a metabolic point of view, the debranching enzyme hydrolyzes the alpha 1,6-glycogen bond to yield glucose-1-phosphate as final product [13]. Because of the metabolic block in the patients, muscle accumulates subsarcolemmal and intermyofibrillar glycogen, leading to dissociation of myofibrils (actin-myosin). The accumulated glycogen has a normal structure and leads to progressive disruption of the myofibrillar architecture [4, 5], and development of muscle weakness.

GSDIII is the third most prevalent muscle glycogenosis following glycogen storage disease type V, GSDV or McArdle disease (OMIM 232600), and glycogen storage disease type II, GSDII or Pompe disease (OMIM 232300), a lysosomal acid maltase deficiency [8–11]. In Pompe disease, there is an abnormal accumulation of glycogen inside the lysosomes of many cell types. In muscle cells, this lysosomal accumulation of glycogen is seen as vacuoles of variable size [9]. Moreover autophagic debris accumulates due to an impaired fusion between autophagosomes and dysfunctional lysosomes [14].

Autophagic flux has never been studied in GSDIII skeletal muscle muscles. However, seminal morphological description of GSDIII human muscle reported that rare structures resembling lysosomes can be observed mixed with glycogen vacuoles in muscle fibers [5].

Recently, a new murine model of GSDIII which faithfully recapitulates the human condition was created, and successfully treated using dual overlapping adeno-associated virus (AAV) derived vectors leading to the restoration of the GDE enzyme activity body-wide [2]. This proof-of-concept may support a future translation of the AAV-based gene therapy approach for GSDIII to the clinic.

In the present study we performed an extensive analysis of morphology and ultrastructure of 30 GSDIII muscle biopsies collected through a large international multicenter collaboration. We describe human muscle morphological phenotype of GSDIII, and we highlight the ultrastructural and protein evidence of increased but dysfunctional autophagy in both human and murine GSDIII skeletal muscles.

## Material and methods

### Patients

This study was approved and performed under the ethical guidelines issued by the different involved institutions and in compliance with the Helsinki Declaration. Informed consent was obtained from all patients. This study did not require ethics approval as no identifying information or patient images were recorded.

Thirty patients of various ethnic backgrounds were included in the present study. In 29 patients GSDIII diagnosis was genetically or enzymatically confirmed. Genetic and enzymatic analysis results were not available for P30; GSDIII diagnosis was therefore considered highly possible based on clinical and histological findings. There were muscle samples from 11 females and 19 males, collected through an international multicentric collaborative study involving 6 countries. Eleven muscle samples came from France, 13 from Japan, 3 from Denmark, 1 from each of Germany, Spain and Sweden. Clinical and morphological findings from patient number 27 (P27) has been previously reported in [18]. The other 29 cases are reported herein for the first time.

Clinical summaries and laboratory features of all patients are provided in Tables 1 and 2. Of note, full clinical information was available for 11 patients. For 19 patients, only short clinical reports addressed to the pathologist were available. All the patients underwent an open muscle biopsy for morphological, immunochemical and biochemical analyses on snap-frozen muscle tissue.

**Table 1:** Clinical and diagnostic features of patients

Patient	Center	Sex	Current Age (y)	Age Onset	Age at biopsy (y)	Biochemistry Debranching enzyme activity (%)	*AGL* (NM_000642.2) Nucleotide /protein change
P1	France	M	79	At birth	55	ND	ND
P2	France	F	58	6 y	35	ND	c.4456del; p.Ser1486Profs*18 (hom)
P3	France	M	40	ND	28	Absent (leucocytes), 0%	c.3216_3217del; p.Glu1072Aspfs*36 (hom)
P4	France	F	85	66 y	59	Reduced (muscle), 30%	c.3652C>T; p.Arg1218* (hom)
P5	France	F	32	18 y	ND	ND	c.3216_3217del; p.Glu1072Aspfs*36 (hom)
P6	France	F	24	9 m	ND	ND	c.364_365dup; p.Pro123Tyrfs*12 (hom)
P7	France	M	61	39 y	40	ND	ND
P8	France	M	60	Childhood	33	ND	c.4322_4323dup; p.Gly1442Lysfs*28 (hom)
P9	France	F	71	50	53	ND	ND
P10	France	M	37	Childhood	6	ND	ND
P11	France	M	28	ND	22	ND	c.3216_3217del; p.Glu1072Aspfs*36 (hom)
P12	Japan	F	38	ND	16	ND	c.371T>A; p.Leu124*(hom)
P13	Japan	M	ND	ND	ND	ND	c.1672dup; p.Thr558Asnfs*4 (hom)
P14	Japan	M	33	11 m	7	ND	c.1003del; p.Ile335Leufs*14 (het) c.1222C>T; p.Arg408*(het)
P15	Japan	M	44	14 y	15	Reduced (muscle), 42%	ND
P16	Japan	F	32	At birth	3	Severely reduced (muscle), 10%	c.3816_3817del; p.Gly1273Asnfs*18 (het), c.4027G>A; p.Glu1343Lys (het)
P17	Japan	M	52	At birth	24	ND	c.853C>T; p.Arg285* (hom)
P18	Japan	F	63	Infancy	38	Severely reduced (muscle), 1%	c.1169_1172del; p.Asn390Ilefs*26 (hom)
P19	Japan	M	35	23 y	23	Severely reduced (muscle), 15%	Negative
P20	Japan	M	64	50 y	53	Severely reduced (muscle), 6%	c.118C>T; p.Gln40*(hom)
P21	Japan	M	63	1 y	56	Severely reduced (muscle), 5%	c.2813-2A>C; p.? (hom)
P22	Japan	M	64	42 y	58	ND	c.1022A>G; p.Tyr341Cys (hom)
P23	Japan	M	52	6 y	48	Reduced (muscle), 25%	c.1672dup; p.Thr558Asnfs*4 (hom)
P24	Japan	F	49	6 y	46	Severely reduced (muscle), 2%	c.1735+1G>T; p.? (hom)
P25	Denmark	F	47	At birth	46	ND	ND
P26	Denmark	M	26	4 y	24	Absent (muscle), 0%	ND
P27	Denmark	M	31	First months	30	ND	c.3179C>A; p.Ser1060* (het), p.Met766fs (het)
P28	Germany	F	59	10 y	49	ND	c.3980G>A; p.Trp1327* (hom)
P29	Sweden	M	71	25 y	50	ND	c.3613C>T; p.Gln1205* (hom)
P30	Spain	M	53	48 y	49	ND	ND

*Abbreviations: F* female, *M* male, *y* years, *m* months, *ND* not determined, *hom* homozygous, *het* heterozygous

**Table 2:** Clinical features of patients

Patients		*n* = 30	
Proportion of female (%)		*n* = 11	33%
Median current age (years)		*n* = 28	52
Median age at biopsy (years)		*n* = 27	38
Onset age	Before 1 years old	7	23,3%
	First decade	8	26,7%
	Second decade	3	10,0%
	Third decade	2	6,7%
	Fourth decade	1	3,3%
	Fifth decade	3	10,0%
	After 50 years old	3	10,0%
	Unknown	3	10,0%
Clinical features	Asymptomatic	1	3,3%
	Muscles disease (fatigability, exercise intolerance, muscle weakness)	21	70,0%
	Cardiac involvement (cardiomegaly, rhythmic abnormalities)	10	33,3%
	Hepatic involvement (hypoglycemia, hepatopathy)	18	60,0%
	Respiratory involvement	3	10,0%
Biopsied muscle	*L. biceps* brachii	10	33,3%
	*L. vastus* (medialis or lateralis)	7	23,3%
	L. Deltoid	5	16,7%
	L. Palmaris	1	3,3%
	Tibial anterior	1	3,3%
	R. Rectus femoris	1	3,3%
	Unknown	5	16,7%

### Muscle morphological studies

Histochemical and histoenzymological analysis was conducted on 30 muscle biopsies. Age at muscle biopsy was available in 27 patients and ranged from 3 years to 59 years (median 38 years). Open muscle biopsies were obtained from different muscles reported in Tables 2 and 3. Histological and histochemical slides were systematically retrospectively analyzed. To describe significant histologic findings, two non-overlapping consecutive representative regions of cryostat sections at 20X magnification stained with hematoxylin and eosin (HE), Periodic Acid Schiff (PAS), and immunostained with SQSTM1/p62 were analyzed. The morphological data were quantified using a scoring system evaluating the number of affected fibers (Table 3). The morphological criteria used for quantification are fully described in Table 4. For conventional histological and histochemical stains, 10 μm thick cryostat sections were stained with hematoxylin and eosin (HE), modified Gömöri trichrome (GT), Periodic acid Schiff technique (PAS), Oil red O, reduced nicotinamide adenine dinucleotide dehydrogenase-tetrazolium reductase (NADH-TR), succinic dehydrogenase (SDH), cytochrome c oxidase (COX), and adenosine triphosphatase (ATPase) preincubated at pH 9.4, 4.63, 4.35. Digital photographs of biopsies were obtained with a Zeiss AxioCam HRc linked to a Zeiss Axioplan Bright Field Microscope and processed with the Axio Vision 4.4 software (Zeiss, Germany).

**Table 3:** Morphologic data

Patient	Sex	Age at biopsy (years)	Biopsied muscle	Light microscopy									Electron microscopy (EM)		
				HE						PAS		Immunohisto-chemistry (IHC)			
				Internalized nuclei	Fibrotic areas	Vacuoles	Ring fibers	Necrosis	Regeneration	Intensity	Positive vacuoles	SQSTM1/p62	Inter-myofibrillar glycogen accumulation	Autophago-somes	Autophagic structures
P1	M	55	L. Deltoid	++	++	+++	+	–	–	++	+++	NP	+	+	–
P2	F	35	L. Deltoid	+	+	+++	++	–	+	+	+++	NP	++	++	–
P3	M	28	L. Deltoid	++	+	+	++	–	–	++	++	NP	+	++	++
P4	F	59	L. Palmaris	++	++	+++	++	–	++	++	+++	NP	+	++	+
P5	F	ND	ND	++	+++	++	++	–	+	++	++	NP	+++	++	+
P6	F	ND	ND	++	++	++	+	–	–	+	++	NP	++	+	++
P7	M	40	L. Deltoid	++	+++	+++	+	–	–	+++	+++	NP	+	+	–
P8	M	33	ND	+++	+	++	++	–	+	++	++	NP	++	++	–
P9	F	53	L. Vastus lateralis	+	++	+	++	–	–	+++	+	NP	+	++	–
P10	M	6	ND	+++	++	++	+	–	–	+	++	NP	++	+	+
P11	M	22	ND	+	+++	++	–	–	–	++	++	+	+	+	+
P12	F	16	L. Biceps brachii	+	++	++	–	–	–	+++	+	+	NP	NP	NP
P13	M	ND	L. Biceps brachii	++	++	++	–	–	–	+	–	+	NP	NP	NP
P14	M	7	L. Biceps brachii	+	+	+	–	–	–	+	–	+	NP	NP	NP
P15	M	15	L. Deltoid	+	+	+	–	–	–	+++	+	+	NP	NP	NP
P16	F	3	L. Biceps brachii	+	++	+++	–	–	–	+++	++	+	NP	NP	NP
P17	M	24	L. Biceps brachii	++	+	+++	–	–	–	+	+	–	NP	NP	NP
P18	F	38	R. Rectus femoris	+++	+++	+++	–	–	–	+	+	+	NP	NP	NP
P19	M	23	L. Biceps brachii	+	+	++	–	–	–	+++	+++	+	NP	NP	NP
P20	M	53	L. Biceps brachii	++	+	+++	–	+	++	+	+	+	NP	NP	NP
P21	M	56	L. Biceps brachii	++	–	++	–	+	+	++	+	+	NP	NP	NP
P22	M	58	L. Biceps brachii	+++	+	+	++	–	–	+++	++	–	NP	NP	NP
P23	M	48	L. Biceps brachii	+++	+	+++	–	–	+	+++	++	–	NP	NP	NP
P24	F	46	L. Vastus medialis	+++	+++	+++	–	–	–	+++	++	NP	NP	NP	NP
P25	F	46	L. Vastus lateralis	+	+++	+++	–	–	+	+++	+++	NP	+	+++	–
P26	M	24	L. Vastus lateralis	+++	++	+++	–	–	+++	+++	+++	NP	+	++	–
P27	M	30	L. Vastus lateralis	+++	+++	+++	–	–	+++	+++	++	NP	+++	+	–
P28	F	49	Tibial anterior	+	+++	+++	–	–	++	+	+++	NP	+	++	–
P29	M	50	L. Vastus lateralis	++	++	++	–	–	+++	+++	++	NP	++	++	–
P30	M	49	L. Vastus lateralis	++	++	+++	+++	–	+	++	+++	NP	NP	NP	NP

ND: not determined; HE: Hematoxylin and eosin; PAS: Periodic acid Schiff. +,++,+++: see Table 4. -: absence. NP: not performed

**Table 4:** Morphologic criteria

			Number of affected fibers
HE	Internalized nuclei	+	< 10%
		++	10–30%
		+++	> 30%
	Fibrotic areas	+	< 5%
		++	5–30%
		+++	> 30%
	Vacuoles	+	< 5%
		++	5–30%
		+++	> 30%
	Ring Fibers	+	< 5%
		++	5–10%
		+++	> 10%
	Necrosis	+	< 2%
		++	2–5%
		+++	> 5%
	Regeneration	+	< 2%
		++	2–5%
		+++	> 5%
PAS	Augmented intensity	+	< 5%
		++	5–30%
		+++	> 30%
	Positive vacuoles	+	< 5%
		++	5–30%
		+++	> 30%
IHC	SQSTM1/p62	+	< 5%
		++	5–30%
		+++	> 30%
EM	Intermyofibrillar glycogen accumulation	+	< 5%
		++	5–30%
		+++	> 30%
	Autophagosomes	+	< 5%
		++	5–30%
		+++	> 30%
	Autophagic structures	+	< 5%
		++	5–30%
		+++	> 30%

For abbreviations see Table 3

Immunohistochemical (IHC) study was performed on sections from available frozen muscle samples of 13 patients. An antibody against SQSTM1/p62 (Anti-Human P62, Progen Biotechnik, Heidelberg, Germany) was used.

Electron Microscopy (EM) studies were performed in 16 samples. Significant ultrastructural findings observed are reported in Table 3 after quantification following the morphological criteria reported in Table 4.

Small muscle specimens were fixed with glutaraldehyde (2.5%, pH 7.4), post fixed with osmium tetroxide (2%), dehydrated and embedded in resin. Longitudinally oriented ultra-thin sections were obtained at different level of deepness from 1 to 3 small blocks and stained with uranyl acetate and lead citrate. Ultra-thin sections of transversally oriented blocks were obtained only for the most significant findings. The grids were observed using a Philips CM120 electron microscope (80 kV; Philips Electronics NV, Eindhoven, The Netherlands) and were photo-documented using a Morada camera (Olympus Soft Imaging System, Hamburg, Germany).

### Western blot (WB) analysis in human muscle biopsies

WB studies were performed in 15 patients (11 from Japan and 4 from France). Frozen skeletal muscles were sliced and solubilized in a buffer containing 0.125 M Tris, 2% SDS, 10% glycerol, 5% 2-mercaptoethanol, pH 6.8. SDS-PAGE was done following Laemmli’s method. An equal amount of protein (30 μg) was separated on 5–20% polyacrylamide gels (FUJIFILM Wako, Neuss, Germany) and transferred onto a PVDF membrane. The membrane was incubated with the following antibodies against (1:1000 dilution unless otherwise stated): LC3 (1:500) (NB100–2220, Novus Biologicals, Abingdon, UK), GAPDH, (1:1000) (ab8245,Abcam, Cambridge, UK), BNIP3, SQSTM1/p62, beclin- (Abcam, Cambridge, UK), and beclin- 1 (#3738 Cell Signaling Technology, clone (D40C5), MA, USA).

### Mouse studies

All mouse studies were performed according to the French and European legislation on animal care and experimentation (2010/63/EU) and approved by the local institutional ethical committee (protocol no. 2016–002). AAV vectors were administered intravenously via the tail vein to 3-month-old male Agl^−/−^ mice and wild-type littermates (Agl^+/+^).

### Western blot (WB) analysis in mouse samples

Snap-frozen skeletal muscles from GDE KO mice were mechanically homogenized in Pierce RIPA Buffer (Thermo Fisher Scientific, Waltham, MA) using lysing matrix tubes (MP Biomedicals, Illkirch-Graffenstaden, France). Protein concentration was determined using the BCA Protein Assay (Thermo Fisher Scientific, Waltham, MA).

SDS-page electrophoresis was performed using a 4–15% gradient polyacrylamide gel. After transfer, the membrane was blocked with Odyssey buffer (Li-Cor Biosciences, Lincoln, NE) and incubated with anti-SQSTM1/p62 (ab56416 Abcam), anti-Tubulin (T9026, Sigma-Aldrich, St Louis, MO), anti-GAPDH (PA1–988, Thermo Fisher), anti-Vinculin (V9131, Sigma-Aldrich). The membrane was washed and incubated with the appropriate secondary antibody (Li-Cor Biosciences) and visualized by Odyssey imaging system (Li-Cor Biosciences).

### Measurement of glycogen content

Glycogen content was measured indirectly in tissue homogenates as the glucose released after total digestion with Aspergillus Niger amyloglucosidase (diastase, Sigma-Aldrich, Saint Louis, MO). Samples were incubated for 5 min at 95 °C and then cooled at 4 °C; 25 μl of amyloglucosidase diluted 1:50 in 0.1 M potassium acetate pH 5.5 were then added to each sample. A control reaction without amyloglucosidase was prepared for each sample. Both sample and control reactions were incubated at 37 °C for 90 min. The reaction was stopped by incubating samples for 5 min at 95 °C. The glucose released was by a glucose assay kit (Sigma-Aldrich, Saint Louis, MO) and the resulting absorbance was acquired on an EnSpire alpha plate reader (Perkin-Elmer, Waltham, MA) at a wavelength of 540 nm.

## Results

### Clinical and laboratory findings

Patient summaries and laboratory features are provided in Table 1. Sanger sequencing of *AGL* gene was performed in 22 patients. Debranching enzyme activity was measured in 16 patients. Clinical features are summarized in Table 2. Patients initially attended a pediatric clinic of metabolic disorders. Data relative to pediatric hospitalization was not available. According to the clinical reports, disease onset was during the first year in 7 patients (23.3%) and before 10 years of age in 8 patients (26.7%). The rest of the patients had a later onset (> 10 years, 50%), (Table 1). Twenty-one patients had muscle involvement (70%), 18 patients had hepatic involvement (60%), 10 patients had cardiac involvement (33.3%), while respiratory involvement was found in 3 patients (10%).

### Morphological findings

**Fig. 1:**
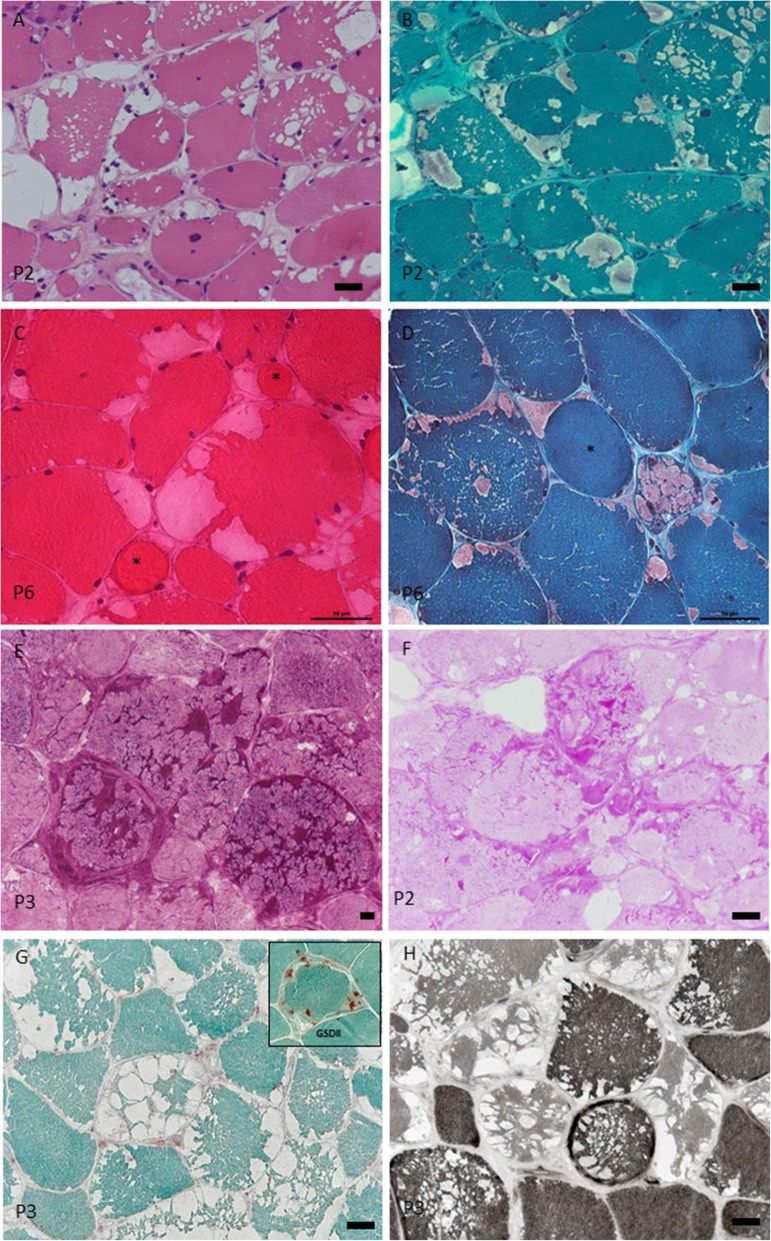
Muscle morphological studies. Light microscopy. GSDIII morphological spectrum. **a.** H&E. Vacuolar myopathy with the majority of fibers harboring multiple and variably sized vacuoles present in both subsarcolemmal and central areas of the fibers. Note the presence of nuclear internalization or centralization and the proliferation of endomysial conjunctive tissue. **b.** mGT. The vacuoles appear filled with a faint eosinophilic material. The vacuoles confer a shabby appearance to the fibers. **c.** HE. Muscle sections showing at least two ring fibers (asterisks). The ring fibers are rounded, atrophic and surrounded by vacuoles suggesting that the myofibrils dissociated from the sarcolemma reorganize themselves with a different orientation. **d.** mGT. Ring fiber surrounded by a subsarcolemmal vacuole (asterisk). **e.** PAS. The section shows intensively stained areas of the cytoplasm corresponding to glycogen-laden vacuoles. The staining is intense also in areas of the cytoplasm without vacuoles. **f.** PAS. Section showing a milder staining intensity and some optically empty vacuoles. **g.** Acid Phosphatase. Normal acid phosphatase reaction demonstrating absence of lysosomal activation. Insert, intense acid phosphatase reaction in muscle from a patient with late onset Pompe disease is seen. **h.** ATPase 9.4 Section showing an equal distribution of the vacuoles in both type 2 (light fibers) and type 2 (dark fibers)

Significant morphologic findings observed with HE and PAS staining for each patient are reported on Table 3. By light microscopy, all patients presented a remarkably stereotyped pathologic picture of a vacuolar myopathy with multiple and variably sized vacuoles visible in a large percentage of muscle fibers (Fig. 1a). The vacuoles appeared mostly optically empty with HE staining (Fig. 1a), and variably filled with faintly stained uneven eosinophilic material with mGT staining (Fig. 1b). The vacuoles were single but more often multiple, lobulated, separated by remnants of cytoplasm, and randomly distributed inside the muscle fibers. In some fibers they were organized giving an embroided aspect to the myocytes (Fig. 1 a, b). Other pathological features included prominent fiber size variation with rounded fibers surrounded by a thick band of endomysial tissue as well as atrophic fibers. No necrotic/regenerating fibers were observed in the muscle biopsies. In 10 cases we observed numerous ring fibers (Fig. 1 c, d). Ring fibers belonged to both fibers type and appeared to be due to vacuole-mediated myofibrillar reorganization leading to loss of docking of the myofibrils from the subsarcolemmal areas.

With PAS staining, the staining for glycogen and glycans, we observed different scenarios. In some biopsies an intense PAS staining was visible throughout the section with affected muscle fibers having variably sized vacuoles filled with PAS positive material (Fig. 1e). In some fibers an association of big vacuoles, appearing purple, with empty vacuoles was seen (Fig. 1f), the latter possibly due to glycogen washed out during sample preparation (Fig. 1c). The vacuoles were intermingled with islands of apparently normal myofibrillar structure. The presence of ring fibers was particularly evident with this technique. Ring fibers often showed a preserved cytoplasm suggesting a secondary reorganization of the fibers trying to get rid of the glycogen. PAS positive material inside the vacuoles was digested by diastase (not shown). Acid phosphatase reaction was normal (Fig. 1g), demonstrating the absence of lysosomal activation. Vacuoles were equally present in both type 1 and type 2 fibers (Fig. 1h). There was no uneven oxidative staining neither type 1 nor type 2 predominance.

#### In conclusion

GSDIII muscle biopsies present with multiple vacuoles variably filled with PAS positive material harboring an embroided aspect.

**Fig. 2:**
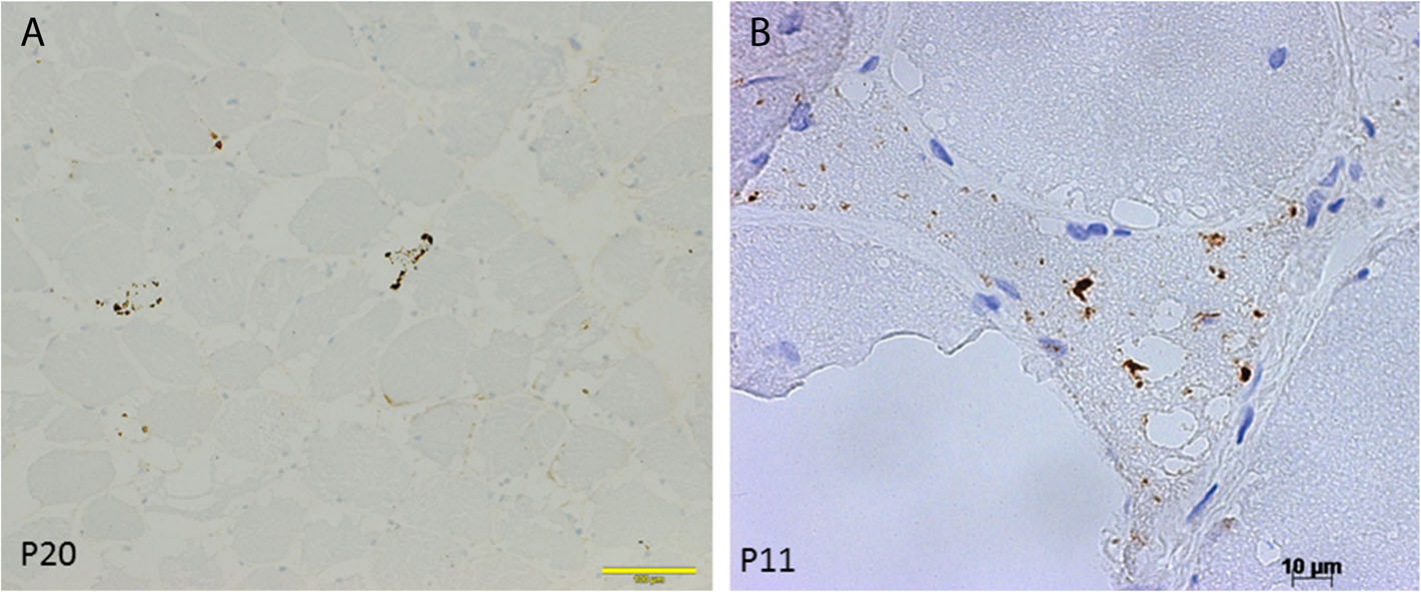
p62/SQTM1 immunohistochemical analysis (immunoperoxidase). **a.** Presence of scattered fibers showing dotty aggregates. **b.** Atrophic fiber showing numerous dotty aggregates present inside the vacuoles, cytoplasm and subsarcolemmal areas

Immunohistochemical studies for SQSTM1/p62 were performed in 13 muscle biopsies (Table 3). Scattered muscle fibers, often atrophic, showing dotty immunoreactive material inside small vacuoles were present in 10 samples (Fig. 2a). Some atrophic fibers harbored numerous small dotty P62 positive inclusions (Fig. 2b). The inclusions were found both inside the vacuoles and in subsarcolemmal areas (Fig. 2b).

### Electron microscopy study

**Fig. 3:**
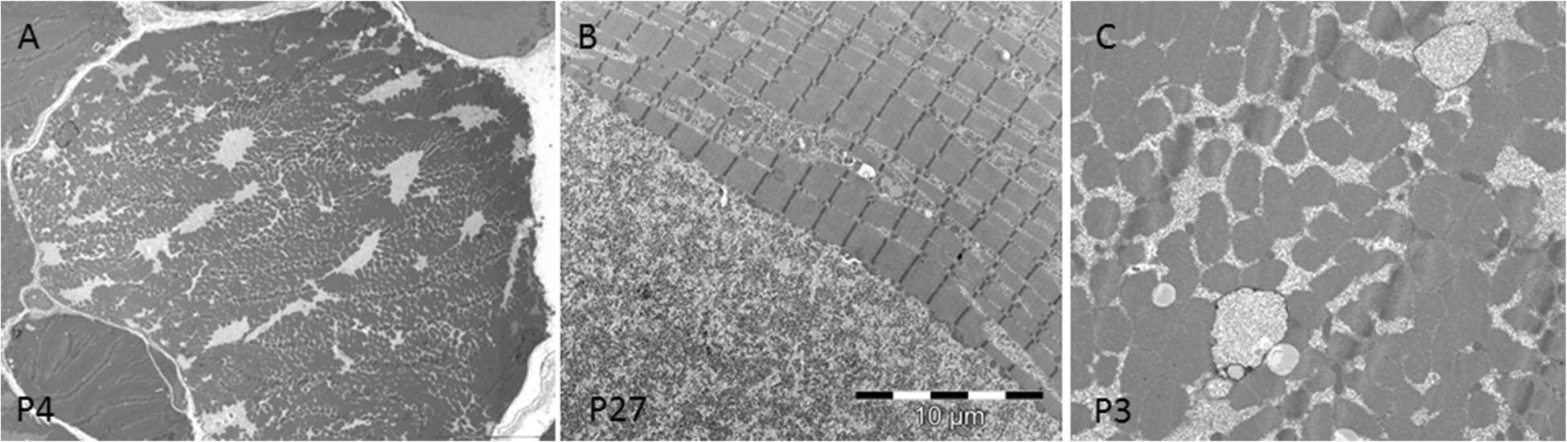
Ultrastructural findings of GSDIII. **a.** Cross section of a vacuolated muscle fibers showing myofibrils dissociated by variable sized pools of clear areas evenly distributed in the cytoplasm and subsarcolemmal areas. **b.** Longitudinal section showing large areas composed of granular material contiguous to the sarcomeric structure. The sarcomeres present a variable width and are sometimes dissociated by accumulated glycogen. **c.** Cross section showing the presence of dissociated myofibrils due to accumulated glycogen. Few rounded sac-like structures filled with glycogen are also observed

Significant ultrastructural findings for each analyzed patient are reported on Table 3. Ultrastructural studies showed the presence of multiple foci of glycogen surcharge variably distributed inside the fibers in all samples. The glycogen was more abundant than normal in intermyofibrillar areas and seemed to progressively store in bigger glycogen deposits (Fig. 3a). In longitudinal sections, we observed large areas of glycogen storage composed by variable dense glycogen granules. The granules had a normal size of about 5–18 nm. We did not observe any filamentous or granular protein material (Fig. 3b) associated with granular glycogen.

Glycogen storage led to progressive dissociation of myofibrils that tended to bifurcate and sometimes, appeared to drown in the glycogen pools (Fig. 3b). Inside the glycogen pools, we often observed mitochondria with a normal size and shape. The overall sarcomeric structure was normal without signs of myofibrillar degeneration. The atrophic fibers were observed in proximity of destroyed bigger fibers.

**Fig. 4:**
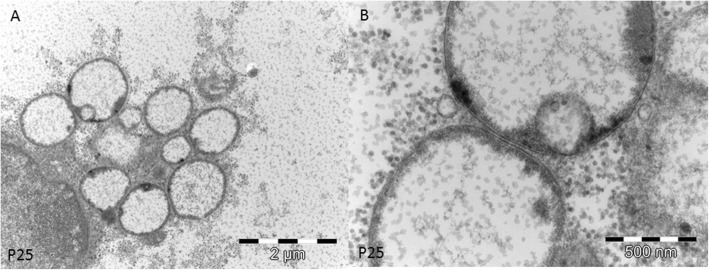
Ultrastructural findings of GSDIII. **a.** Group of sac-like structures lined by membranes containing finely granular material. **b.** At higher magnification the sac-like structures have a double membrane and correspond to an autophagosome

In all samples the presence of sac-like structures spanning 2–3 myofibrils was observed (Fig. 3c). These structures contained glycogen granules morphologically identical to free intermyofibrillar glycogen (Fig. 3c). These sacs were found isolated or in groups either near the nuclei or in proximity of the accumulated free glycogen (Fig. 4a).

**Fig. 5:**
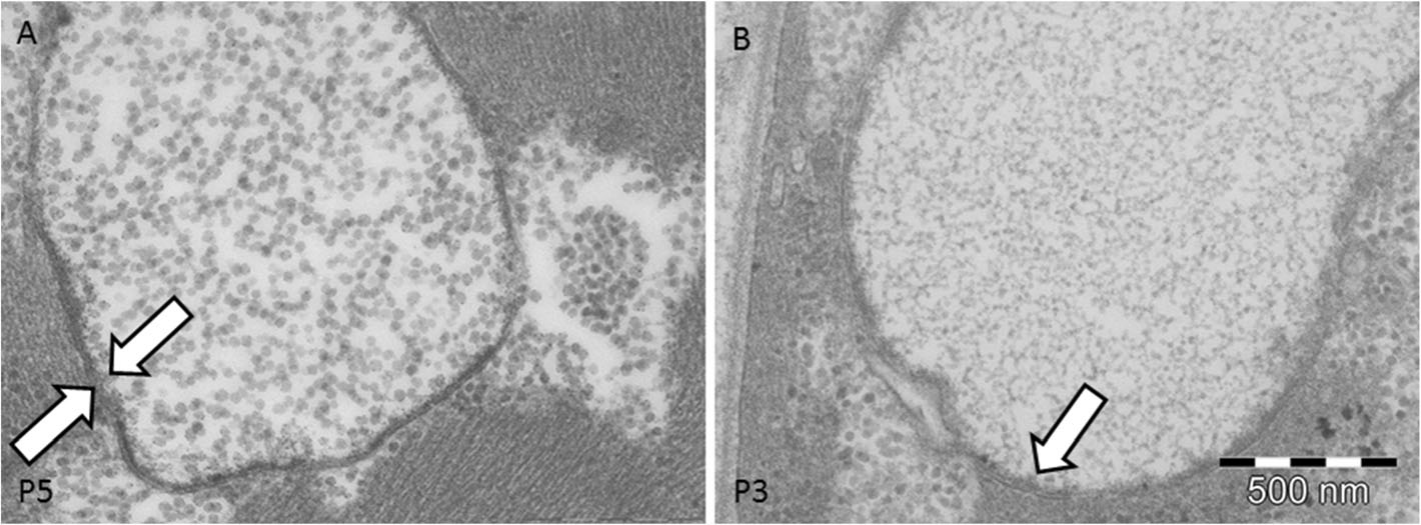
Ultrastructural findings of GSDIII. **a.** Autophagosome delimited by a thicker external and a thinner internal membrane. **b.** Autophagosome containing uneven finely granular material

**Fig. 6:**
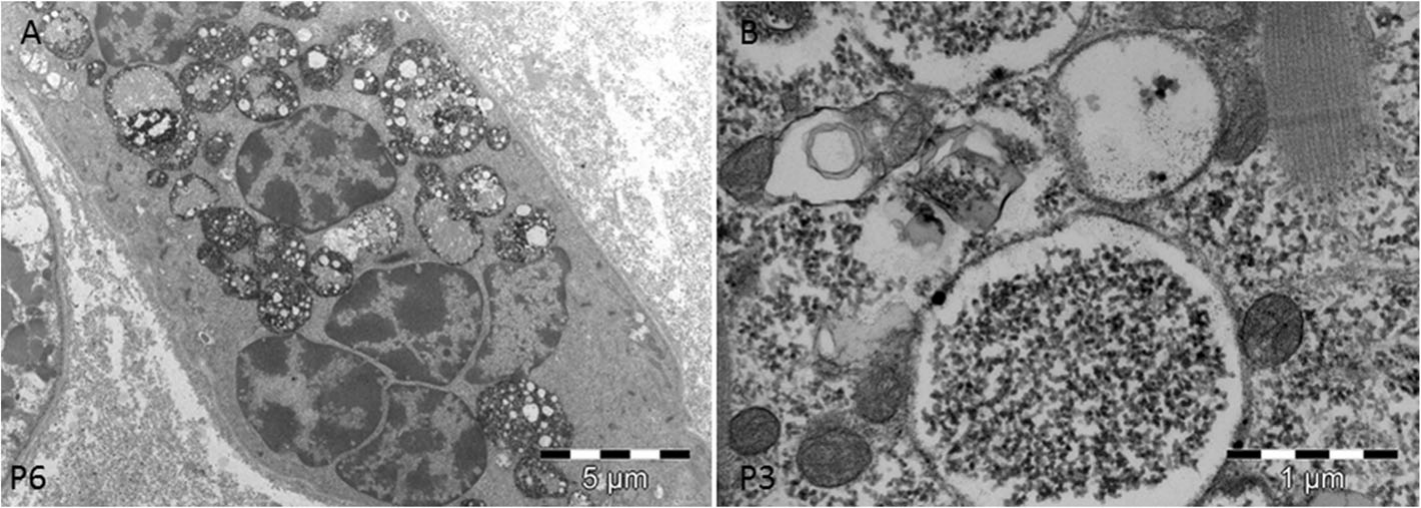
Ultrastructural findings of GSDIII. **a.** Atrophic fiber containing nuclear clumps, surrounded by completely degenerated myofibrils and autophagic material. **b.** Prominent autophagic material containing vacuoles, cellular debris and more intensely osmiophilic dotty structures

At higher magnification, the glycogen laden vacuoles appeared to have a double membrane with a denser external layer (Fig. 4 a and b) and an uneven, irregular internal layer (Fig. 5 a and b). Outside these vacuoles, normal granular glycogen and normal myofibrils were present (Fig. 5a). These structures, harboring two membranes and a clear intermembrane space, corresponded to autophagosomal structures at different stage of maturation, clearly different from lysosomes which usually display a dense intermembrane space. The content of autophagosomes had variable density, likely related to the stage of autophagic digestion (Fig. 5) a and b. In very atrophic fibers, autophagosomes were associated with clusters of nuclei or mixed with different autophagic structures (Fig. 6a). Autophagosomes were also associated with normally sized mitochondria and were found near the remnants of myofibrils drowned in glycogen pools. In 6 samples, we observed variable sized and shaped autophagic material such as pseudo-myelinic structures and remnant of organelles (Fig. 6b).

#### In conclusion

all samples showed large accumulations of normally structured glycogen and double membrane structures corresponding to autophagosomes.

### Monitoring autophagy in GSDIII patient muscle

To investigate whether autophagy was involved in GSDIII muscle pathology, we studied muscle biopsies from patients with GSDIII and controls for autophagy core protein expression. Formation of autophagosomes requires phosphatidylinositol-3-kinase (PI3K) complex, which includes the vacuolar protein sorting 34 (VPS34/PIK3C3), VPS15, beclin-1 and ATG14L. This complex generates phosphatidylinositol-3-phosphate (PI3P), which allows the recruitment of the autophagy conjugation system for the autophagosome formation. Ubiquitinated cargos are recruited to the autophagophore membrane via the interaction of the autophagy receptor SQSTM1/p62 with LC3II. Decreased levels of SQSTM1/p62 are observed upon autophagy induction, whereas increased levels are a marker of autophagy flux blockage [1].

**Fig. 7:**
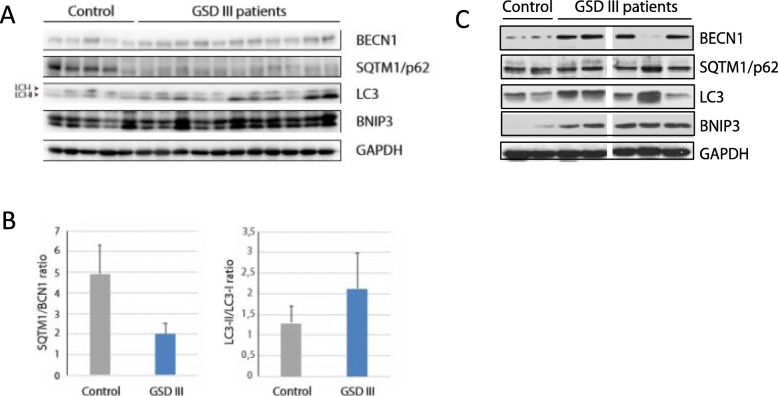
Autophagic flux studies in human muscle biopsies. **a.** Western blot for BECN1, LC3I/II and SQSTM1/p62 in Japanese GSDIII patients (P12, P13, P14, P15, P16, P17, P18, P19, P20, P21, P22) and controls. **b**. Quantification of SQSTM1/p62 decrease and beclin-1 increase levels in GSDIII patients versus controls. Accordingly, an increase in the lipidated form of LC3; LC3II was measured in patients compared to control. **c.** Western blot for beclin-1, total LC3 and SQSTM1/p62 in five French GSD3 patients (P1, P5, P7, P8 and P 11) and controls

Using immunoblotting we quantified beclin-1, LC3I/II and SQSTM1/p62 in GSDIII patients and control muscle biopsies (Fig. 7 a and b). We observed both a decrease of SQSTM1/p62 levels and an increase of beclin-1 levels in GSDIII patients. This decreased SQSTM1/p62/beclin-1 ratio (4.9 ± 1.4) in control versus 2.0 ± 0.5 in GSDIII patients) measured in the same protein extracts strongly suggests an enhanced autophagy. Accordingly, an increase in the lipidated form of LC3; LC3II was measured in patients compared to control (Fig. 7c). There was no difference in relative expression of the BH3 family protein BNIP3, which plays a role in recognizing damaged mitochondria by facilitating docking of autophagosomes through binding to LC3 [7] between patients and control. Altogether, our results demonstrate an increase of autophagy flux in GSDIII patient muscles.

### Autophagy studies in GSDIII mouse muscle samples

**Fig. 8:**
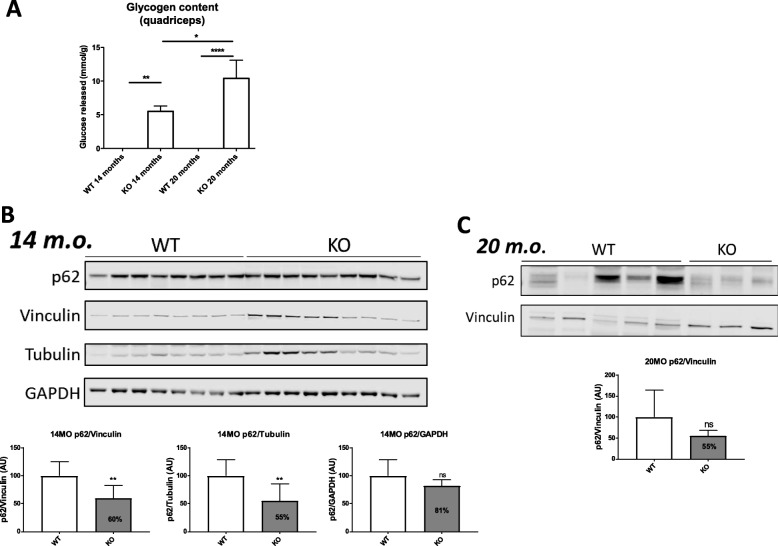
Autophagic flux studies in GSDIII mouse tissues. **a.** Augmented glycogen content in both GDE KO mice of 14 and 20 months**. b.** SQSTM1/p62 protein by immunostaining of total protein lysates of quadriceps tissues showing variable levels of SQSTM1/p62 proteinold mice. **c.** p62/SQSTM1 protein by immunostaining of total protein lysates of quadriceps tissues in 20 months GSDIII mice showing slightly decreased level of p62

A new murine model of GSDIII, which recapitulates human phenotype including muscle weakness (PMID: 29396266) demonstrated metabolic impairment and reduced muscle strength [18]. Lack of debranching enzyme (AGL) resulted in accumulation of glycogen in quadriceps muscle of this mouse model (Fig. 8a). Given the ultrastructural defects and autophagy upregulation observed in human patients, the mouse model was used to assess the autophagy dysfunction in 14- and 20-month-old mice. We analyzed the levels of SQSTM1/p62 protein by immunostaining of total protein lysates of quadriceps tissues. p62/SQSTM1 can directly bind to LC3 and be degraded by the autophagy machinery. The muscle tissues of 14 months old mice revealed variable levels of p62 protein. Overall, we observed a slightly decreased p62 levels both in 14- and 20-months old mice without major difference between the groups (Fig. 8) b-c.

## Discussion

This is the first international multicenter study investigating muscle biopsy features in a large cohort of 30 GSDIII patients. We aimed at demonstrating pathophysiologic pathways that could prove feasible targets for gene therapy.The study limitations were the retrospective design, the scarce amount of muscle samples to perform more extended immunohistochemical and protein studies in the cohort, and limited access to updated clinical information for some patients.

All patients presented with a stereotyped and consistent light microscopy morphological picture of a vacuolar myopathy characterized by the presence of multiple and variably sized vacuoles conferring an embroided aspect to the muscle fibers (Fig. 1). The vacuoles were filled with PAS positive material or optically empty. The last features could be related to technical preparation. Different muscles were biopsied in the cohort, but that didn’t affect the uniform histological picture, and both type 1 and 2 fibers were equally vacuolized. All the biopsies were taken for diagnostic purposes at a median age of 38. Although it was beyond the objective of this study, interestingly, no correlation between clinical severity and degree of vacuolar myopathy was found in this study. For example, muscle biopsy from P4 showed a severe morphological phenotype in association with mild proximal muscle weakness rated 4 at the MRC manual testing.

GSDIII is a bi-phasic disorder where hepatomegaly and hypoglycemia are the initial symptoms. Increased muscle fatigability appears during adolescence, followed by permanent muscle weakness in adulthood [3], likely due to glycogen-mediated muscle damage exceeding a threshold at which point the weakness becomes clinically evident.

Our morphologic analysis allowed wondering toward the dynamic of formation of the vacuolar lesions. Due to deficiency of GDE enzyme, glycogen cannot be degraded and begin to accumulate throughout the fiber, mechanically dissociating myofibrils (Fig. 1a). In some fibers, we observed a loss of docking of the myofibrils to the sarcolemma, due to subsarcolemmal accumulation of glycogen. Fibrosis was evident in some cases, without signs of concurrent necrosis or regeneration. It is possible that this process has a slow dynamic with muscle weakness appearing only when fibrosis starts to appear (Fig. 1 a, and b).

Another peculiarity that we observed in 12 muscle biopsies was the presence of ring fibers (Fig. 1) c and d. The ring fibers are constituted by peripheral myofilaments placed perpendicular to the other myofibers of the same bundle [6]. This disposition, to some eyes, mimics the back of a ‘spiral’ notebook. Ring fibers, albeit considered a non-specific histological finding, have been described in different chronic myopathies (e.g., myotonic dystrophy, limb-girdle dystrophy, inclusion body myositis), and tenotomy [17]. While previous studies suggested the involvement of hypertonic muscles state, aberrant synthesis of myofibril proteins, or cytoskeletal defects, the natural cause of ring fiber formation remains elusive. Recently, an actin regulator CAP2, which is crucial for the exchange of α-actin isoforms during myofibril differentiation has been characterized [10]. In CAP2 mutant mice, this α-actin switch was delayed and coincided with the onset of a myopathy characterized by type IIB ring fibers and motor dysfunction. In our study, we did not find a correlation between the type of fibers and ring fibers [10]. In GSDIII, ring fibers could be related to a mechanical phenomenon where massive vacuolization provoke myofibrils detachment and successive reorientation of some bundles. Studies on patient-derived myoblasts and myotubes could answer this question. The presence of ring fibers in a background of a vacuolar myopathy should alert myopathologists to consider a GSDIII.

GSDIII is the third most frequent cause of glycogenosis with fixed muscle weakness after glycogen storage disease type V, or McArdle disease (OMIM 232600), and glycogen storage disease type II or Pompe disease (OMIM 232300). GSDII is an autosomal recessive disorder caused by mutations in the acid a-glucosidase (*GAA*) gene on chromosome 17q25.2–q25.3 [8]. Affected individuals accumulate excessive glycogen in the lysosomes and cytoplasm of several tissues and show to have autophagy dysregulation [14].

The histologic picture found in our patients differs substantially from that of GSDII and in particular from the late-onset form of Pompe disease (LOPD) [8]. GSDIII muscles do not show lysosomal dysfunction with Acid Phosphatase reaction (Fig. 1e). The embroided disposition is peculiar to GSDIII and all patients in our series invariantly showed a vacuolar myopathy. Vacuoles have a less specific shape and disposition in LOPD (personal observation) and were found in around 60% of patient with LOPD [15].

Ultrastructural studies in GSDIII demonstrated something unexpectedly in all samples the presence of double membrane structures filled with glycogen (Figs. 3, 4, 5 and 6) dispersed in large deposit of free cytoplasmic glycogen. In some samples, we also found prominent autophagosomes. The presence of dispersed and rare lysosomes in GSDIII muscle biopsy has previously been documented [5]. The images showed in our study differ substantially from lysosomes, being membrane-bound cytoplasmic organelles with an electron dense intermembrane space [16]. The intermembrane space was persistently clear in GSDIII sac structures (Fig. 6), indicating that they are autophagosomes. Autophagosome formation is the first step of autophagy, an evolutionarily mechanism in which macromolecule and organelles are initially degraded before being fused to lysosomes for final degradation or recycling [9]. The autophagic pathway plays a crucial role in skeletal muscle homeostasis, providing a finely tuned system which mediates protein degradation and organelle removal [12]. A dysfunctional autophagy has been described in numerous myopathies. For example, autophagy failure yields massive autophagic buildup in glycogen storage disease type II (GSDII) [14].

In order to confirm a possible autophagic dysregulation dysfunction, we first performed immunohistochemistry for SQSTM1-positive protein aggregates that is a recognized and validated marker of impaired autophagic flux [15]. In 10 of our samples we observed the presence of scattered muscle fibers, often atrophic, showing dotty immunoreactive material inside small vacuoles (Fig. 2a). Some fibers harboring numerous small dotty P62 positive inclusions (Fig. 2b) displayed atrophic features. We then studied muscle biopsies of GSD-III patients and control for autophagy core protein expression. We showed an evident autophagy dysregulation in muscle biopsies from our patients indicated by a decreased SQSTM1/p62/BECN1 ratio (4.9+/− 1.4) in control versus 2.01+/− 0.49 in GSD-III patients (Fig. 7). Enhanced autophagy could be interpreted as an attempt of the muscle fiber to get rid of the accumulated glycogen or damaged organelles and sarcomeres.

Very recently a GSDIII mouse model have been created by Vidal et al. [22]. This model faithfully recapitulated the clinical phenotype of GSDIII in man and showed muscle morphological features like that observed in our series. GSDIII mice were treated using a dual AAV vector expressing the GDE transgene under the control of a liver-specific promoter. With this strategy, treated animals showed improved glycemia and clearance of liver and muscular glycogen with force amelioration.

We tested the same parameter of autophagy flux in mice with GSDIII deficiency. Although there was a tendency toward autophagy alteration similar to what have been observed in our samples, this was not statistically significant. Of interest, in the previous paper from *Sun* et al. [20], the authors studied whether the overexpression of the lysosomal enzyme GAA could rescue the phenotype of GSDIII. However, despite achieving supraphysiological levels of GAA activity in most tissues, in GSDIII mice this approach had only a limited and transient effect on muscle performance. A significant reduction of glycogen accumulation was only observed in liver, without any effect on glycemia or hepatomegaly. These results were consistent with data obtained in vitro with GSDIII myoblasts treated with recombinant GAA enzyme [13].

Our study demonstrate altered autophagy in GSDIII muscle confirms the rationale of autophagy modulation, e.g. rapamycin [23], or combination with diet [2], to favor glycogen removal through the lysosomal pathway.

## Conclusions

In conclusion, the work presented here describes the muscle morphological phenotype of GSDIII, identifies autophagy increase as a possible compensatory mechanism to contrast the glycogen surcharge, and supports the rationale that autophagy modulation can be a therapeutic option for patients with GSDIII.

## Data Availability

All data are available.

## Abbreviations

AAV: Adeno-associated virus
ATPase: Adenosine triphosphatase
COX: Cytochrome c oxidase
EM: Electron microscopy
GDE: Glycogen debranching enzyme
GSDII: Glycogen storage disorder type II (Pompe disease)
GSDIII: Glycogen storage disorder type III
HE: Hematoxylin and eosin
IHC: Immunohistochemical
LOPD: Late onset Pompe disease
mGT: modified Gömöri trichrome
PAS: Periodic Acid Schiff
WB: Western blot
